# Accuracy of Predicted Genomic Breeding Values in Purebred and Crossbred Pigs

**DOI:** 10.1534/g3.115.018119

**Published:** 2015-05-26

**Authors:** André M. Hidalgo, John W. M. Bastiaansen, Marcos S. Lopes, Barbara Harlizius, Martien A. M. Groenen, Dirk-Jan de Koning

**Affiliations:** * Wageningen University, Animal Breeding and Genomics Centre, Wageningen, the Netherlands 6700 AH; † Swedish University of Agricultural Sciences, Department of Animal Breeding and Genetics, Uppsala, Sweden 750 07; ‡ Topigs Norsvin Research Center, Beuningen, the Netherlands 6640 AA

**Keywords:** GenPred, shared data resource, across-population, genomic selection, multi-population, reproduction traits, within-population

## Abstract

Genomic selection has been widely implemented in dairy cattle breeding when the aim is to improve performance of purebred animals. In pigs, however, the final product is a crossbred animal. This may affect the efficiency of methods that are currently implemented for dairy cattle. Therefore, the objective of this study was to determine the accuracy of predicted breeding values in crossbred pigs using purebred genomic and phenotypic data. A second objective was to compare the predictive ability of SNPs when training is done in either single or multiple populations for four traits: age at first insemination (AFI); total number of piglets born (TNB); litter birth weight (LBW); and litter variation (LVR). We performed marker-based and pedigree-based predictions. Within-population predictions for the four traits ranged from 0.21 to 0.72. Multi-population prediction yielded accuracies ranging from 0.18 to 0.67. Predictions across purebred populations as well as predicting genetic merit of crossbreds from their purebred parental lines for AFI performed poorly (not significantly different from zero). In contrast, accuracies of across-population predictions and accuracies of purebred to crossbred predictions for LBW and LVR ranged from 0.08 to 0.31 and 0.11 to 0.31, respectively. Accuracy for TNB was zero for across-population prediction, whereas for purebred to crossbred prediction it ranged from 0.08 to 0.22. In general, marker-based outperformed pedigree-based prediction across populations and traits. However, in some cases pedigree-based prediction performed similarly or outperformed marker-based prediction. There was predictive ability when purebred populations were used to predict crossbred genetic merit using an additive model in the populations studied. AFI was the only exception, indicating that predictive ability depends largely on the genetic correlation between PB and CB performance, which was 0.31 for AFI. Multi-population prediction was no better than within-population prediction for the purebred validation set. Accuracy of prediction was very trait-dependent.

Genomic selection has been widely implemented in dairy cattle breeding when the aim is to improve performance of purebred animals (Berry *et al.* 2009; VanRaden *et al.* 2009; Hayes *et al.* 2009b). In pigs and poultry, however, the final product is a crossbred animal. This may affect the efficiency of methods that are currently implemented for dairy cattle. In pig breeding, multiple sire and dam lines are used, with a minimum of two lines (typically for crossbred sows) and often additional sire lines to produce a three-way or four-way cross finisher pig (Merks and De Vries 2002; Lutaaya *et al.* 2001).

Selection based on genomic estimated breeding values (GEBV) for purebreds (PB) using phenotypes on crossbreds (CB) is expected to increase the response to selection observed in CB compared to the situation in which only PB phenotypes are used. This increased response is expected when the genetic correlation between the PB and CB trait is less than 1, especially when the genetic correlation is 0.7 or less (Dekkers 2007). Genetic correlations between PB and CB performance vary and can be considerably less than 1 (Lutaaya *et al.* 2001; Zumbach *et al.* 2007; Cecchinato *et al.* 2010). Adding CB individuals to the training data is very expensive because, besides genotyping, it also requires additional identification and individual recording of target traits. Breeding companies are not inclined to make these investments unless there is evidence that predictions yield greater gains and higher accuracies. Simulation studies have shown that the response to selection is greater when PB animals are selected based on CB performance and that accuracy of prediction is high (Dekkers 2007; Ibánez-Escriche *et al.* 2009; Kinghorn *et al.* 2010; Toosi *et al.* 2010; Zeng *et al.* 2013). There is, however, a lack of studies using real data. The number of genotyped CB is not yet large enough to test the superiority of training on CB for PB selection. A first step toward finding the optimal genomic selection scenario for pigs is to determine predictive ability (accuracy), in real data, of GEBV for CB pigs based on PB genomic and phenotypic data. This will show how CB performance responds to the current practice of selection on GEBV in PB pigs.

Recently, accuracies of within-population genomic prediction in pigs have been reported (Cleveland *et al.* 2010; Forni *et al.* 2011; Christensen *et al.* 2012; Tusell *et al.* 2013; Badke *et al.* 2014). These studies have shown that all traits had more than zero predictive ability within population in a variety of pig breeds using different methods. It has also been shown that using genomic information generally increased the accuracy of prediction compared to using only pedigree information (Forni *et al.* 2011; Christensen *et al.* 2012; Tusell *et al.* 2013). Using multi-population training might be a way to increase the accuracy of prediction further. This is especially relevant to enable genomic selection for small populations when a closely related breed, or the same breed from another country, is added to the training set (Lund *et al.* 2014). An unresolved question is how to obtain accurate predictions from multi-population datasets. The effectiveness of a multi-population genomic evaluation depends on many factors, *e.g.*, differences in allele frequency and consistency of linkage disequilibrium (LD) between quantitative trait loci (QTL) and single nucleotide polymorphism (SNP), which could reduce the accuracy of prediction (Wientjes *et al.* 2013), whereas the larger reference population would potentially improve the accuracy.

The objective of our study was to determine predictive ability (accuracy) in CB pigs using real PB genomic and phenotypic data. The outcome is a first step toward determining the optimal genomic selection scenario to select PB for CB performance. As in cattle, studying accuracy of prediction for multi-population datasets is important for species in which population size imposes upper limits to the training population size. Therefore, a second objective was to compare the predictive ability of SNPs when training is done in either single or multiple populations in pigs.

## Materials and Methods

### Data

Genotypes were available from sows with own-performance information of three pig populations born from 2005 through 2012: 1070 Dutch Landrace-based (DL) sows from 19 farms; 1389 Large White-based (LW) sows from 14 farms; and 287 individuals from an F1 cross between these two commercial lines (DL sire/LW dams) originating from three farms. The genotyped CB animals had no specific family structure and the majority of them were not offspring of the genotyped PB animals, *i.e.*, a number of generations separated PB and CB. The 287 CB animals were offspring from 76 sires and 170 dams. Four female reproduction traits were analyzed: age at first insemination (AFI); total number of piglets born (TNB); litter birth weight (LBW); and litter variation (LVR). AFI consisted of the age at the second estrus, which was the time that the first insemination was performed. TNB was the sum of all piglets born alive and stillborn. LBW was the sum of individual birth weights of all piglets born in the same litter. Finally, LVR consisted of the standard deviation (SD) of individual birth weight of the piglets from the same litter.

The PB and CB sows that were selected for genotyping have phenotypic records from multiple parities on multiple traits and have a large genetic contribution to future descendants. All PB sows were breeding animals from nucleus farms, whereas the CB sows belonged to farms where combined crossbred and pure line selection (CCPS) is applied. There was no strong selection for first parity performance in the genotyped sows, reducing any possible bias in TNB and LBW due to culling after first parity.

Deregressed estimated breeding values (DEBV) were used as response variable for each trait undergoing study. The estimated breeding values (EBV) were deregressed for each trait separately using the methodology proposed by Garrick *et al.* (2009). DEBVs, instead of EBVs, were used to compute the GEBV accuracy because this removes the influence of the parents’ EBVs and rescales the EBV according to its accuracy, *i.e.*, the DEBV of the animals reflect their genetic merit. Ostersen *et al.* (2011) have shown that using DEBVs rather than EBVs for genomic prediction yields higher GEBV accuracies. The number of animals and records used to estimate the EBVs are in Table 1. The EBV of each animal was obtained from the routine genetic evaluation by Topigs Norsvin using MiXBLUP (Mulder *et al.* 2012) in a multi-trait model (including all measured reproduction traits). The genetic evaluation was done across lines with phenotypes from the different populations treated as the same trait. A fixed line effect was included in the model for estimating EBVs. In multi-population prediction scenarios, this line effect was added back to the random additive genetic effect after estimating the EBVs, and subsequently, the line effect was again included in the genomic prediction model. Adding back the line effect allows the differences of the level of EBV between-population to be maintained in the data. Therefore, in the genomic prediction step, the mean differences between populations are still present, and this allows SNP effects (that differ in allele frequencies between lines) to explain these differences between lines.

**Table 1:** Number of phenotypes on crossbreds and purebreds that were used to estimate the breeding values

Trait	No.	DL	LW	F1	Total
AFI	Records	304,853	203,933	190,828	699,614
	Animals	304,853	203,933	190,828	699,614
TNB	Records	1,483,099	910,349	864,551	3,257,999
	Animals	344,583	223,088	211,117	778,788
LBW	Records	158,546	152,722	7051	318,319
	Animals	46,221	43,403	2093	91,717
LVR	Records	158,167	146,500	7037	311,704
	Animals	46,124	42,350	2083	90,557

AFI, age at first insemination; TNB, total number of piglets born; LBW, litter birth weight; LVR, litter variation.

The model for obtaining the EBVs for AFI included genetic line and herd-year-season as fixed effects and an additive genetic effect (animal) as random effect. For TNB, the fixed effects were genetic line, parity, interval between weaning and pregnancy (days), whether more than one insemination procedure was performed (yes or no), and herd-year-season. The random effects consisted of service sire, a permanent effect to account for the repeated observations of a single sow, and an additive genetic effect (animal). EBVs for LBW were obtained with a model that included genetic line, parity number, TNB, and herd-year-season as fixed effects and a permanent effect and an additive genetic effect (animal) as random effects. The model used for LVR was similar to the one used for LBW, except that TNB was removed. The reliabilities per animal, needed for deregression, were extracted from the genetic evaluation based on the methodology of Tier and Meyer (2004). The heritabilities (h^2^) used for deregression were estimated via restricted maximum likelihood (REML) using a pedigree-based relationship matrix and were also obtained from the routine genetic evaluation. The h^2^ of the traits were 0.30 for AFI, 0.11 for TNB, 0.38 for LBW, and 0.14 for LVR. The genomic h^2^ of the DEBVs were estimated via REML using ASREML 3.0 (Gilmour *et al.* 2009).

Sows were genotyped using the Illumina PorcineSNP60 BeadChip (Ramos *et al.* 2009). SNPs with GenCall <0.15, unmapped SNPs, and SNPs located on either the X or the Y chromosome, according to the Sscrofa10.2 assembly of the reference genome (Groenen *et al.* 2012), were excluded. Quality control was performed in all populations simultaneously, which involved excluding SNPs with call rate <0.95, minor allele frequency <0.01, and strong deviations of Hardy-Weinberg equilibrium (*χ^2^* > 600). After quality control, 42,139 SNPs remained out of the initial 64,232 SNPs. Individuals with missing genotype frequency >0.05 were also removed. Missing genotypes of the remaining animals were imputed using BEAGLE 3.3.2 (Browning and Browning 2007).

### Statistical analyses

GEBVs were computed based on the genomic best linear unbiased prediction method (GBLUP). GBLUP uses a genomic relationship matrix (**G**) instead of the numerator relationship matrix (**A**). The **G** matrix contains genomic kinship indicating relatedness between animals and was used for prediction in all scenarios with the model:

y = 1μ + Zg + e,

where **y** is the vector of DEBVs, *μ* is the overall mean, **g** is the vector of random additive genetic effects assumed to be ∼N(**0**, **G**${\text{σ}}_{a}^{2}$), **Z** is a design matrix allocating **g** to **y**, and **e** is a residual with heterogeneous variance due to differences in reliabilities of the DEBVs (Garrick *et al.* 2009). In predictions where the training set contained more than one population, the fixed line effect present in the model for estimating EBVs was also included in the GBLUP model as a fixed effect.

The **G** matrix for within-population prediction was built according to VanRaden (2008), which was computed as $G=\mathrm{ZZ\prime}/ 2\sum p_{i}q_{i}$, where **Z** is a matrix of centered genotypes and $p_{i}$ and $q_{i}$ are the allelic frequencies of the *i^th^* marker based on observed genotypes. In predictions where the training set contained more than one population, the **G** matrix was built according to Chen *et al.* (2013), accounting for differences in allele frequencies between populations.

We used ASREML 3.0 (Gilmour *et al.* 2009) to predict the GEBVs, with the **G** matrix entered as a user-defined matrix. Animals assigned to the prediction set had their DEBVs removed before predicting GEBV.

All scenarios were also analyzed using the **A** matrix, which contains the average additive genetic relationships of the animals based on the pedigree (PED-BLUP). The model for these analyses was similar to the GBLUP one; however, the **g** vector of the random additive genetic effect was assumed to be ∼N(**0**, **A**${\text{σ}}_{a}^{2}$).

Genetic correlations between PB and CB performance were estimated for the four traits. We used records for DL, LW, and F1 animals born from 2005 through 2012 (Supporting Information, Table S1). Genetic correlations were estimated in bivariate analyses using REML in ASREML 3.0 (Gilmour *et al.* 2009). The effects of bivariate models were the same as those used to obtain the EBVs (see above); however, to estimate genetic correlations, PB performance and CB performance were treated as different traits (Falconer 1952), which in matrix notation is:

$$\left[\begin{array}{c} y_{1}\\ y_{2} \end{array}\right]=\;\left[\begin{array}{cc} Z_{1} & 0\\ 0 & Z_{2} \end{array}\right]\left[\begin{array}{c} g_{1}\\ g_{2} \end{array}\right]+\left[\begin{array}{c} e_{1}\\ e_{2} \end{array}\right]$$

where **y_i_** is the vector of observations with *i* being 1 for purebred and 2 for crossbred data, **Z_i_** is the incidence matrix for **g_i_**, which is a vector of random additive genetic effects. The additive genetic variance is expressed as:

$$\text{var}\left[\begin{array}{c} g_{1}\\ g_{2} \end{array}\right]=G_{0}⊗A$$

where **A** is the numerator relationship matrix and **G_0_** is a 2×2 covariance matrix with the purebred and crossbred variances in the diagonals and the covariances in the off-diagonals.

### Scenarios and accuracy of prediction

Seventeen scenarios were investigated that can be divided into four groups according to composition of the training and validation data sets as follows:

- Scenarios 1–3: Training and validation data were subsets from the same population, DL, LW, and F1, respectively, *i.e.*, prediction was within-population. These scenarios determine how well the within-population prediction performs for the different traits.
- Scenarios 4–7: Same as scenarios 1–3 but the remaining PB population(s) was/were added to the training data, *i.e.*, prediction was multi-population. These scenarios determine whether adding data from a different PB population to the training data would increase the accuracy compared to the within-population prediction.
- Scenarios 8–11: One PB population was used for training to predict the other PB population. F1 data were not used in these scenarios, *i.e.*, prediction was across breeds. These scenarios determine how well across-population predictions would perform.
- Scenarios 12–17: PB populations were used for training and CB animals were used for validation. These scenarios determine how well CB genetic merit can be predicted from PB data alone, and whether inclusion of more than one parental PB population increases the accuracy.

The accuracy of prediction was estimated as the correlation between the GEBV/EBV and the DEBV of the validation set animals for GBLUP/PED-BLUP. Prediction bias was calculated by regressing the validation variables (DEBV) on the prediction variables (GEBV/EBV). Accuracies were the average of 20 random training-validation populations in scenarios 1–7, 9, 11, 13, 15, and 17. For scenarios 1–7, we randomly set aside part of the genotyped animals (N = 50) and used those in a later step to determine the accuracy of prediction. These 50 were not included in the training for those scenarios. In scenarios 9, 11, 13, 15, and 17, not all the available animals were used for training. Subsets of the training populations were sampled such that the same number of animals was used from each population per trait undergoing study. Any differences in accuracies would then be due to the different populations used, and not to differences in the number of animals. Scenarios 8, 10, 12, 14, and 16 only had one estimate of accuracy because all the animals were used in the training population to maximize prediction accuracy of animals in another population.

## Results

Estimates of genomic h^2^ of the DEBVs across traits and populations ranged from 0.04 to 0.58 (Table 2). Estimates of pedigree-based h^2^ of the DEBVs across traits and populations ranged from 0.03 to 0.78 (Table S2). The genomic and pedigree-based heritabilities were similar in general. Genetic correlations between PB performance and CB performance for the four traits undergoing study ranged from 0.31 for AFI to 0.90 for LBW (Table 3).

**Table 2:** Estimated genomic heritability (h^2^) of the deregressed estimated breeding values across traits and populations under study

	Heritability (SE)		
Trait	DL	LW	F1
AFI	0.18 (0.04)	0.07 (0.02)	0.64 (0.12)
TNB	0.04 (0.01)	0.05 (0.01)	0.12 (0.05)
LBW	0.58 (0.05)	0.57 (0.04)	0.43 (0.12)
LVR	0.21 (0.03)	0.11 (0.02)	0.17 (0.07)

DL, Dutch Landrace; LW, Large White; F1, cross between DL and LW; AFI, age at first insemination; TNB, total number of piglets born; LBW, litter birth weight; LVR, litter variation.

**Table 3:** Genetic correlations between purebred and crossbred performance for the four traits undergoing study

Trait	Genetic Correlation (SE)
AFI	0.31 (0.02)
TNB	0.88 (0.01)
LBW	0.90 (0.05)
LVR	0.88 (0.06)

AFI, age at first insemination; TNB, total number of piglets born; LBW, litter birth weight; LVR, litter variation.

Accuracies for within-population predictions for scenarios 1–3 ranged from 0.22 to 0.72 for GBLUP and from 0.21 to 0.64 for PED-BLUP across the four traits and different training sets, indicating a modest to good predictive ability (Table 4). The regression coefficient of the GEBV/EBV on the DEBV for scenarios 1–3 ranged from 1.03 to 1.70 for GBLUP and from 0.90 to 2.21 for PED-BLUP.

**Table 4:** GEBV accuracies from within-population prediction using GBLUP and PED-BLUP (scenarios 1–3)

			N Training			N Validation			Accuracy*^a^* (SD)		Slope*^b^*	
Trait	Scenario	r^2^	DL	LW	F1	DL	LW	F1	GBLUP	PED-BLUP	GBLUP	PED-BLUP
AFI	1	0.45	1017	—	—	50	—	—	0.26 (0.16)	0.25 (0.15)	1.21	1.13
	2	0.45	—	1339	—	—	50	—	0.22 (0.09)	0.21 (0.19)	1.25	1.08
	3	0.33	—	—	237	—	—	50	0.37 (0.09)	0.30 (0.12)	1.04	0.90
TNB	1	0.45	1016	—	—	50	—	—	0.26 (0.12)	0.25 (0.12)	1.70	1.90
	2	0.49	—	1333	—	—	50	—	0.24 (0.15)	0.25 (0.15)	1.24	1.50
	3	0.40	—	—	231	—	—	50	0.40 (0.11)	0.35 (0.14)	1.52	2.21
LBW	1	0.78	1020	—	—	50	—	—	0.64 (0.09)	0.58 (0.06)	1.08	1.06
	2	0.80	—	1335	—	—	50	—	0.72 (0.06)	0.64 (0.07)	1.03	1.05
	3	0.77	—	—	236	—	—	50	0.40 (0.11)	0.39 (0.13)	1.10	1.27
LVR	1	0.50	1019	—	—	50	—	—	0.50 (0.11)	0.40 (0.10)	1.04	1.03
	2	0.53	—	1335	—	—	50	—	0.46 (0.09)	0.39 (0.15)	1.05	1.17
	3	0.49	—	—	235	—	—	50	0.34 (0.09)	0.33 (0.11)	1.03	1.19

r^2^ is the mean reliability of deregressed estimated breeding values from the training population. SD, standard deviation, DL, Dutch Landrace; LW, Large White; F1, cross between DL and LW; AFI, age at first insemination; TNB, total number of piglets born; LBW, litter birth weight; LVR, litter variation. Scenario 1, within-population prediction for DL; scenario 2, within-population prediction for LW; scenario 3, within-population prediction for F1.

a Estimate obtained by 20-random training-validation populations.

b Regression coefficient of the GEBV/EBV on the DEBV.

For multi-population prediction of PB populations (scenarios 4 and 5) the accuracies ranged from 0.18 to 0.67, whereas for multi-population prediction (two PB + one CB) of the CB population (scenarios 6 and 7) the accuracies ranged from 0.17 to 0.45 for GBLUP and from 0.32 to 0.42 for PED-BLUP. When predicting PB (scenarios 4 and 5; Table 5), the addition of the other PB population resulted in lower accuracies for all four traits in comparison to within-population prediction for GBLUP. When predicting CB (scenarios 6 and 7; Table 5), the addition of PB populations resulted in lower accuracies for AFI and TNB but higher accuracies for LBW and LVR. The regression coefficient of the GEBV/EBV on the DEBV for scenarios 4 and 5 ranged from 0.86 to 1.18 for GBLUP, whereas for scenarios 6 and 7 it ranged from 0.80 to 3.11 for GBLUP and from 0.97 to 5.00 for PED-BLUP. Accuracies and regression coefficients of the EBV on the DEBV were not computed for PED-BLUP for scenarios 4 and 5 because the other PB population to be added is not related according to the pedigree.

**Table 5:** GEBV accuracies from multi-population prediction using GBLUP and PED-BLUP (scenarios 4–7)

			N Training			N Validation			Accuracy*^a^* (SD)		Slope*^b^*	
Trait	Scenario	r^2^	DL	LW	F1	DL	LW	F1	GBLUP	PED-BLUP	GBLUP	PED-BLUP
AFI	4	0.45	1017	1389	—	50	—	—	0.20 (0.13)	—	1.18	—
	5	0.45	1067	1339	—	—	50	—	0.18 (0.13)	—	1.11	—
	6	0.41	1067	1389	237	—	—	50	0.17 (0.09)	0.32 (0.12)	1.91	1.86
	7	0.41	237	237	237	—	—	50	0.27 (0.11)	0.35 (0.11)	3.11	2.17
TNB	4	0.47	1016	1383	—	50	—	—	0.21 (0.15)	—	1.12	—
	5	0.47	1066	1333	—	—	50	—	0.23 (0.17)	—	1.11	—
	6	0.44	1066	1383	231	—	—	50	0.31 (0.18)	0.34 (0.11)	1.69	3.06
	7	0.44	231	231	231	—	—	50	0.37 (0.14)	0.33 (0.12)	3.02	5.00
LBW	4	0.79	1020	1385	—	50	—	—	0.51 (0.13)	—	0.86	—
	5	0.79	1070	1335	—	—	50	—	0.67 (0.07)	—	1.09	—
	6	0.78	1070	1385	236	—	—	50	0.45 (0.11)	0.37 (0.09)	0.80	0.97
	7	0.78	236	236	236	—	—	50	0.41 (0.15)	0.37 (0.11)	1.03	1.10
LVR	4	0.52	1019	1385	—	50	—	—	0.38 (0.12)	—	0.99	—
	5	0.52	1069	1335	—	—	50	—	0.41 (0.12)	—	1.11	—
	6	0.51	1069	1385	235	—	—	50	0.44 (0.10)	0.40 (0.14)	1.22	1.59
	7	0.51	235	235	235	—	—	50	0.38 (0.12)	0.42 (0.08)	1.33	1.88

r^2^ is the mean reliability of deregressed estimated breeding values from the training population. SD, standard deviation, DL, Dutch Landrace; LW, Large White; F1, cross between DL and LW; AFI, age at first insemination; TNB, total number of piglets born; LBW, litter birth weight; LVR, litter variation. Scenario 4, multi-population prediction for DL; scenario 5, multi-population prediction for LW; scenario 6, multi-population prediction for F1; scenario 6, multi-population prediction for F1 with a reduced number of purebred animals.

a Estimate obtained by 20 random training-validation populations.

b Regression coefficient of the GEBV/EBV on the DEBV.

GEBV accuracy of across-breed prediction, *i.e.*, predicting genetic merit of one PB from a different PB population, performed poorly for AFI and TNB (Table 6); accuracies were not significantly different from zero (*P* > 0.05). Accuracies for LBW and LVR ranged from 0.13 to 0.26 across the different training sets for GBLUP. The regression coefficient of the GEBV on the DEBV for AFI and TNB ranged from −0.71 to 1.37, whereas for LBW and LVR it ranged from 0.70 to 1.40. Accuracies and regression coefficients of the EBV on the DEBV were not computed for PED-BLUP because the two PB populations are not related according to the pedigree.

**Table 6:** GEBV accuracies from across-population prediction using GBLUP (scenarios 8–11)

			N Training			N Validation			Accuracy (SD)	Slope*^b^*
Trait	Scenario	r^2^	DL	LW	F1	DL	LW	F1	GBLUP	GBLUP
AFI	8	0.45	1067	—	—	—	1389	—	−0.05	−0.57
	9	0.45	711	—	—	—	1389	—	−0.04 (0.01)*^a^*	−0.71
	10	0.45	—	1389	—	1067	—	—	−0.02	−0.27
	11	0.45	—	711	—	1067	—	—	−0.02 (0.03)*^a^*	−0.43
TNB	8	0.45	1066	—	—	—	1383	—	0.05	1.01
	9	0.45	693	—	—	—	1383	—	0.04 (0.01)*^a^*	1.37
	10	0.49	—	1383	—	1066	—	—	0.03	0.56
	11	0.49	—	693	—	1066	—	—	0.00 (0.02)*^a^*	0.00
LBW	8	0.78	1070	—	—	—	1385	—	0.26	0.83
	9	0.78	708	—	—	—	1385	—	0.23 (0.04)*^a^*	0.83
	10	0.80	—	1385	—	1070	—	—	0.22	0.73
	11	0.80	—	708	—	1070	—	—	0.16 (0.03)*^a^*	0.65
LVR	8	0.50	1069	—	—	—	1385	—	0.17	0.70
	9	0.50	705	—	—	—	1385	—	0.15 (0.03)*^a^*	0.75
	10	0.53	—	1385	—	1069	—	—	0.20	1.40
	11	0.53	—	705	—	1069	—	—	0.13 (0.04)*^a^*	1.22

r^2^ is the mean reliability of deregressed estimated breeding values from the training population. SD, standard deviation, DL, Dutch Landrace; LW, Large White; F1, cross between DL and LW; AFI, age at first insemination; TNB, total number of piglets born; LBW, litter birth weight; LVR, litter variation. Scenario 8, across-population prediction for LW; scenario 9, across-population prediction for LW with a reduced number of DL animals; scenario 10, across-population prediction for DL; scenario 11, across-population prediction with a reduced number of LW animals.

a Estimate obtained by 20-random training-validation populations.

b Regression coefficient of the GEBV on the DEBV.

Accuracy of prediction in scenarios 12–17 that predicted genetic merit of CB using PB parental populations as training data performed poorly for AFI (Table 7); accuracies were not significantly different from zero for both GBLUP and PED-BLUP (*P* > 0.05). For the other three traits, TNB, LBW, and LVR, however, predictive ability was observed. Accuracies ranged from 0.11 to 0.31 for GBLUP and from 0.08 to 0.22 for PED-BLUP. The regression coefficient of the GEBV/EBV on the DEBV for AFI ranged from −1.14 to −0.15 for GBLUP and from 0.15 to 0.95 for PED-BLUP, whereas for TNB, LBW, and LVR it ranged from 0.48 to 3.82 for GBLUP and from 0.53 to 7.76 for PED-BLUP.

**Table 7:** GEBV accuracies from prediction of crossbred genetic merit from purebred training data using GBLUP and PED-BLUP (scenarios 12–17)

			N Training			N Validation			Accuracy (SD)		Slope*^b^*	
Trait	Scenario	r^2^	DL	LW	F1	DL	LW	F1	GBLUP	PED-BLUP	GBLUP	PED-BLUP
AFI	12	0.45	1067	1389	—	—	—	287	−0.07	0.09	−0.75	0.81
	13	0.45	356	356	—	—	—	287	−0.03 (0.06)*^a^*	0.04 (0.06)*^a^*	−0.40	0.53
	14	0.45	1067	—	—	—	—	287	−0.02	0.07	−0.15	0.95
	15	0.45	711	—	—	—	—	287	−0.02 (0.05)*^a^*	0.05 (0.04)*^a^*	−0.24	0.67
	16	0.45	—	1389	—	—	—	287	−0.07	0.06	−1.14	0.73
	17	0.45	—	711	—	—	—	287	−0.06 (0.05)*^a^*	0.01 (0.05)*^a^*	−0.95	0.15
TNB	12	0.47	1066	1383	—	—	—	281	0.20	0.21	1.51	3.02
	13	0.47	347	347	—	—	—	281	0.18 (0.09)*^a^*	0.22 (0.05)*^a^*	3.82	7.76
	14	0.45	1066	—	—	—	—	281	0.18	0.19	2.29	3.62
	15	0.45	693	—	—	—	—	281	0.18 (0.04)*^a^*	0.19 (0.04)*^a^*	3.15	4.71
	16	0.49	—	1383	—	—	—	281	0.13	0.10	1.17	2.23
	17	0.49	—	693	—	—	—	281	0.11 (0.04)*^a^*	0.08 (0.04)*^a^*	1.72	3.06
LBW	12	0.79	1070	1385	—	—	—	286	0.31	0.14	0.62	0.54
	13	0.79	354	354	—	—	—	286	0.18 (0.05)*^a^*	0.11 (0.05)*^a^*	0.52	0.60
	14	0.78	1070	—	—	—	—	286	0.26	0.10	0.65	0.53
	15	0.78	708	—	—	—	—	286	0.22 (0.04)*^a^*	0.14 (0.04)*^a^*	0.64	0.73
	16	0.80	—	1385	—	—	—	286	0.22	0.11	0.55	0.63
	17	0.80	—	708	—	—	—	286	0.17 (0.03)*^a^*	0.08 (0.05)*^a^*	0.48	0.59
LVR	12	0.52	1069	1385	—	—	—	285	0.27	0.15	0.90	0.91
	13	0.52	353	353	—	—	—	285	0.21 (0.08)*^a^*	0.13 (0.07)*^a^*	1.16	1.44
	14	0.50	1069	—	—	—	—	285	0.31	0.11	1.18	0.84
	15	0.50	705	—	—	—	—	285	0.28 (0.04)*^a^*	0.14 (0.05)*^a^*	1.24	1.25
	16	0.53	—	1385	—	—	—	285	0.15	0.11	0.74	1.33
	17	0.53	—	705	—	—	—	285	0.11 (0.04)*^a^*	0.12 (0.05)*^a^*	0.75	2.43

r^2^ is the mean reliability of deregressed estimated breeding values from the training population. SD, standard deviation, DL, Dutch Landrace; LW, Large White; F1, cross between DL and LW; AFI, age at first insemination; TNB, total number of piglets born; LBW, litter birth weight; LVR, litter variation. Scenario 12, purebreds predicting F1 animals; scenario 13, purebreds predicting F1 animals with a reduced number of purebred animals; scenario 14, DL predicting F1 animals; scenario 15, DL predicting F1 animals with a reduced number of DL animals; scenario 16, LW predicting F1 animals; scenario 17, LW predicting F1 animals with a reduced number of LW animals.

a Estimate obtained by 20-random training-validation populations.

b Regression coefficient of the GEBV/EBV on the DEBV.

## Discussion

Accuracies of genomically predicted breeding values in CB and PB pigs were estimated for four female reproduction traits in 17 scenarios to optimize the use of genomic data for crossbred animals. We have used DEBVs as a response variable with a moderate to high mean reliability (ranging from 0.33 to 0.80) for the different traits and populations. The SD of the accuracies in scenarios in which we had replicates of training validation populations varied according to the type of prediction (within, multi-, across, or PB to CB). Within-population and multi-population predictions showed higher SDs because the relationship between training and validation in each replicate could substantially vary due to different degrees of relationships within a population. For across-population and PB to CB predictions, the relationship between training and validation populations was naturally lower; therefore, in each replicate there was less variation.

### Within-population prediction

LBW and LVR showed generally higher accuracies than AFI and TNB. This difference between traits may occur due to the lower reliability of the DEBV for AFI and TNB, which lowers the accuracy when the number of observations is preset. Another possibility is that there are non-additive genetic effects (*e.g.*, dominance, epistasis) affecting AFI and TNB more, whereas LBW and LVR may be regulated mainly by an additive action of the genes. Therefore, the importance of non-additive effects needs to be further investigated. Even with the low number of genotyped CB pigs, all traits showed predictive ability within the CB. Therefore, a greater number of genotyped CB should increase these accuracies. In general, GBLUP outperformed PED-BLUP across populations and traits, which is mainly a result of a better estimation of relationships among individuals by the markers. Similar results have also been reported in other studies using pigs (Forni *et al.* 2011; Tusell *et al.* 2013). The regression coefficients of the GEBV/EBV on the DEBV for both GBLUP and PED-BLUP were, in general, close to 1, indicating that the predictions were not severely biased, except for TNB, where some of the them deviated considerably from 1.

The level of accuracy found here is concordant with those found in other studies on pigs (Cleveland *et al.* 2010; Forni *et al.* 2011; Christensen *et al.* 2012; Tusell *et al.* 2013; Badke *et al.* 2014). In these studies, as well as in ours, many traits and breeds were studied and within-population prediction always had predictive ability. One of the studies (Tusell *et al.* 2013) also studied TNB for two PB populations and their F1 cross and also found that prediction within the F1 cross has greater accuracy than within-PB prediction. They argued that this might be caused by the structure and effective sample size of the populations undergoing study. Accuracies found by Christensen *et al.* (2012) were not statistically different between single-step BLUP (SS-BLUP) and GBLUP, but both were higher than pedigree-based prediction and GBLUP was shown to be more biased. The advantage of using SS-BLUP was an increase of accuracy for non-genotyped animals. Because our aim was to predict genotyped animals, we studied accuracies of prediction using GBLUP.

### Multi-population prediction

Adding data from a different PB population to the training data (scenarios 4 and 5) decreased the accuracy of prediction compared with within-population predictions (scenarios 1–3) for GBLUP. Adding data from the two PB populations to the CB training data (scenarios 6 and 7) had different results depending on the trait. LBW and LVR that had high genetic correlation between PB and CB performance had an increase in accuracy, whereas for AFI that had a low genetic correlation there was a decrease in accuracy. TNB had a high genetic correlation; however, the accuracy also decreased, which was unexpected.

If traits are genetically very different (low genetic correlation between PB and CB), then adding more animals with the other trait to the training is not expected to increase the accuracy. When the trait is the same, however (high genetic correlation), including more animals with the other trait (PB *vs.* CB) is expected to increase the accuracy. Besides having a high genetic correlation between the traits, the additional animals also need to have some (genomic) relationship to the validation animals. In addition to a low genetic correlation between PB and CB performance, the degradation of accuracy might result from differences in non-additive effects.

For PED-BLUP, adding the two parental PB populations in the training also had different results depending on the trait. AFI and LBW had an increase in accuracy, whereas TNB and LVR had a slight decrease in accuracy. The regression coefficient of the GEBV/EBV on the DEBV estimated to investigate bias for scenarios 4–7 was, in general, close to 1, indicating that the predictions did not suffer from a large bias, except for AFI and TNB in scenarios 6 and 7. For these traits, whenever the PB parental populations were used as training and CB used as the validation set, the regression coefficient of the GEBV/EBV on the DEBV indicated that the estimates were severely biased.

A review regarding multi-population prediction in cattle (Lund *et al.* 2014) has shown that combining populations, in general, increases the accuracy of prediction when the breeds are the same but from different countries, to a lesser degree when the breeds are closely related, and has little or no benefit when the breeds are distantly related. Another study (Hayes *et al.* 2009a) has reported slightly higher accuracies when using multi-population prediction compared to within-population prediction in dairy cattle. Chen *et al.* (2013) used Angus and Charolais steers to determine the accuracy of prediction with GBLUP for within-population and multi-population predictions. In their study, accuracies did not always increase, suggesting that noise was being added to the predictions. The maximum increment in accuracy that they obtained was 0.05, whereas a decrement of 0.07 was also obtained, which is within the same range as the differences observed in the current study. These studies showed that adding another PB population to the training data in cattle did not necessarily increase the accuracy of prediction, similar to our current results in pigs.

De Roos *et al.* (2009), using simulated data, also showed that increasing the size of the training data by adding animals from a different population does not always increase the accuracy. An increase in accuracy higher than within-population was only found when the populations were closely related, when marker density was high, or when the size of the initial within-population training data set was small. In our case, the number of markers was reasonable and in some scenarios the size of the within-population training data set was small, but we still did not have a great increase in accuracy of prediction. This suggests that the marker density might not be sufficient to have similar LD levels between QTL and markers in the different populations that are mixed. The genetic distance between the populations was probably an important factor that limited the benefit of adding training data from other populations.

### Across-population prediction

Some predictive ability was observed when predicting across populations for LBW and LVR, whereas for AFI and TNB all the accuracies were null. Increasing the size of the training population slightly improved the accuracies of prediction, on average by 0.05. Greater accuracies were found when DL predicted LW genetic merit, rather than the other way around (scenario 9 *vs.* scenario 11). The regression coefficients of the GEBV on the DEBV for scenarios 8–11 were, in general, close to 1 for LBW and LVR, indicating that the predictions did not suffer from much bias. For AFI and TNB, however, regression values greatly deviated from 1, sometimes with negative values, which we attribute to the very low accuracies we found.

In a study by Harris *et al.* (2008), the prediction across Holstein-Friesian and Jersey cattle breeds was also investigated. Predictions were not accurate, ranging from −0.1 to 0.3 for 25 traits. In another study, Hayes *et al.* (2009a) predicted the GEBV of Jersey animals using a Holstein population as training data and vice versa, resulting in accuracies ranging from −0.06 to 0.23 for five traits. Both studies report results that were very similar to ours that ranged from −0.05 to 0.26.

The simulation study by De Roos *et al.* (2009) indicated that across-population prediction was substantially less accurate than within-population or multiple-population prediction. These lower accuracies were due to differences in marker–QTL LD phase between the populations. A marker may be in LD with QTL in a given population, but it is not necessarily in LD with those QTL in the other population, resulting in poor predictions for the other population. These simulation results suggested that, for our analyses, a higher marker density would be required. However, results of Veroneze *et al.* (2014) show that with the same 60K porcine SNP panel, the density of SNPs is high enough to obtain reasonable levels of LD. This would predict that our SNP panel should be able to capture marker effects across breeds.

### Using purebred training data to predict crossbred genetic merit

Using only the PB population(s) to predict the CB genetic merit with GBLUP has some predictive ability for TNB, LBW, and LVR, whereas all the accuracies for AFI were null. Increasing the size of the training data by adding another PB population increased the accuracy for TNB and LBW, whereas for AFI and LVR it did not. However, when we increased the size of the training population by adding more animals of the same PB population, the accuracies usually increased. The accuracy of prediction for predicting CB animals based on PB animals appears to depend largely on the genetic correlation between PB and CB performance. As our results demonstrate, the greater the genetic correlation, the higher the chances of having any or more predictive ability. AFI, for which the genetic correlation between PB and CB was very poor, had zero accuracy of prediction showing that selection on PB is expected to have no effect on CB genetic merit.

For PED-BLUP, the accuracies were, in general, lower than for GBLUP, especially for LBW and LVR. Adding the second PB population in the training slightly increased the accuracy of prediction.

The greater accuracies found for TNB, LBW, and LVR when training with DL rather than LW population can be explained by the slightly greater relationship between DL and F1 populations than between LW and F1. This higher relationship is specific for the animals included in this study. The F1 animals that were genotyped are more closely related to the DL animals that were genotyped than to the LW animals that were genotyped.

To test the impact of the relationship between training and validation populations on the accuracy, we split the training data into the 50% of animals that are MOST related to the validation set and the 50% that are LEAST related to the validation set (Table S3, Table S4). For AFI, TNB, and LBW, using the 50% MOST related animals resulted in greater accuracies, whereas for LVR it did not. This indicates that if CB animals have closer genomic relationships to the PB animals used as training, then higher accuracies for scenarios 12–17 could generally be expected.

In cattle, Harris *et al.* (2008) used PB populations (Holstein-Friesian and Jersey) to predict the genomic breeding values of a cross between these two breeds. They used data from 4500 sires genotyped for approximately 44K SNPs. Their results show that using the two breeds as the training population increased the accuracy by 5–10% compared to using only one of the breeds to predict the cross. The actual level of accuracy was not reported in their study. Our results were similar to theirs for TNB, LBW, and LVR, where the genetic correlation between PB and CB performance is close to 1, but not for AFI.

Results indicate that using a PB population to predict CB genetic merit can generate reasonable predictions. This, however, is not consistent for all traits. Although these results do not reflect the actual practice of genomic selection in pig breeding, they do provide an estimate of the accuracy of genomic prediction between CB and PB populations using real data. The results make a strong case for the genotyping and recording of CB animals, at least for a subset of traits where genetic correlations are away from 1.

The low genetic correlation between PB and CB performance for AFI was also found in another study (Nagyné-Kiszlinger *et al.* 2013). They have reported values of 0.28 and 0.39 for Hungarian Large White and Hungarian Landrace with their reciprocal cross. Possible reasons for this low genetic correlation were reported: 1) genes that affect the trait might be different among populations; 2) this trait is affected by non-additive effects or environmental factors due to different management of PB and CB animals (Nagyné-Kiszlinger *et al.* 2013). One clear environmental factor that probably reduces the genetic correlation of AFI between PB and CB is the use of batch farrowing systems in the production environment of CB sows. Suppression of estrus is used to synchronize the heat of the CB gilts, which impacts the measurement of the trait and leads to these low correlations.

Standardized EBVs were used; therefore, a bias would possibly be introduced during deregression due to different reliabilities between breeds (Garrick *et al.* 2009). Additional sources for potential bias affecting the SNP effect estimates are the differences in the population mean of the breeds. The differences in the mean between populations were remedied by reintroducing the line effect after deregression. To test the impact of deregression on bias, we investigated all 17 scenarios for the trait AFI by analyzing phenotypes, which are not standardized, instead of DEBVs. The correlation between the accuracies obtained by the two different approaches was 0.92, with a mean regression coefficient of the GEBV on the phenotype of 0.70. This correlation shows that using the phenotypes has good agreement with the accuracies calculated using DEBVs; therefore, any bias due to the process of standardization and deregression is expected to be limited.

The reasonable accuracy for PB predicting CB genetic merit shows that in a current typical breeding program, selection in the PB does result in a phenotypic response in CB. AFI was an exception in our study, because the genetic correlation between PB and CB performance was very low.

Further studies to compare the accuracy of genomic selection of PB for CB performance are needed. Other genomic models including breed-specific effects of SNP alleles or dominance (Ibánez-Escriche *et al.* 2009; Zeng *et al.* 2013) were described and were found to outperform an additive model only in specific cases, *e.g.*, with high dominance levels or when the number of SNPs is small relative to the size of the training population. Using these more complex models or a multiple-trait model (Christensen *et al.* 2014) with real data is needed.

In conclusion, there was predictive ability for purebred population(s) predicting crossbred genetic merit using an additive model in the populations studied when PB and CB traits have high genetic correlation. For practical implementation, estimation of genomic breeding values of PB animals for CB performance needs to be further studied with models that take into account the crossbred nature of training data. Multi-population prediction was no better than within-population prediction for PB populations. Accuracy of prediction was shown to be very trait-dependent; hence, for the utility of data from other breeds in the application of genomic selection, each trait needs to be studied separately and no generalizations should be made. Finally, real data accuracies were lower than what simulation studies have reported.

## 

## Supplementary Material

Supporting Information
