# Supplementary material for: Accuracy of Predicted Genomic Breeding Values in Purebred and Crossbred Pigs
Source: G3 (Bethesda). 2015 May 26;5(8):1575–83. doi: 10.1534/g3.115.018119 (PMC4528314; doi:10.1534/g3.115.018119)
Supplement: Supporting Information [file supp_5_8_1575__index.html]

Accuracy of Predicted Genomic Breeding Values in Purebred and Crossbred Pigs — Supporting Information 

# Accuracy of Predicted Genomic Breeding Values in Purebred and Crossbred Pigs

## Supporting Information for Hidalgo *et al.*, 2015

**Files in this Data Supplement:**

- Supporting Information - Tables S1-S4 and Files S1-S4 (PDF, 144 KB)
- Table S1 - Number of records and individuals used to estimate genetic correlations for purebred and crossbred performance for the four traits under study. (PDF, 74 KB)
- Table S2 - Estimated pedigree-based heritability (h2) of the deregressed estimated breeding values across traits and populations under study. (PDF, 74 KB)
- Table S3 - GEBV accuracies from prediction of crossbred genetic merit from purebred training data using GBLUP (scenarios 12-17) - MOST 50% related animals between training and validation populations. (PDF, 75 KB)
- Table S4 - GEBV accuracies from prediction of crossbred genetic merit from purebred training data using GBLUP (scenarios 12-17) - LEAST 50% related animals between training and validation populations. (PDF, 75 KB)
- File S4 - G matrix for all individuals across populations. (PDF, 103 KB)
- File S1 - Deregressed breeding values for the four traits under study for DL population. (.xls, 139 KB)
- File S2 - Deregressed breeding values for the four traits under study for F1 population. (.xls, 55 KB)
- File S3 - Deregressed breeding values for the four traits under study for LW population. (.xls, 173 KB)
