# Supplementary material for: Accuracy of Predicted Genomic Breeding Values in Purebred and Crossbred Pigs
Source: G3 (Bethesda). 2015 May 26;5(8):1575–83. doi: 10.1534/g3.115.018119 (PMC4528314; doi:10.1534/g3.115.018119)
Supplement: Supporting Information [file supp_g3.115.018119_018119SI.pdf]

G3 · Genes | Genomes | Genetics

Accuracy of predicted genomic breeding values in purebred and crossbred pigs

André M Hidalgo[*,§,1,2] , John WM Bastiaansen[*], Marcos S Lopes[*,†], Barbara Harlizius[†], Martien AM Groenen[*], Dirk-Jan de Koning[§]

[*] Wageningen University, Animal Breeding and Genomics Centre, Wageningen, the Netherlands, 6700 AH.

[§] Swedish University of Agricultural Sciences, Department of Animal Breeding and Genetics, Uppsala, Sweden, 750 07.

[†] Topigs Norsvin, Beuningen, the Netherlands, 6640 AA.

[1] **Present address:** Animal Breeding and Genomics Centre, Wageningen University, building nr. 107, Droevendaalsesteeg 1, 6708 PB Wageningen, the Netherlands.

[2] **Corresponding author:** Animal Breeding and Genomics Centre, Wageningen University, building nr. 107, Droevendaalsesteeg 1, 6708 PB Wageningen, the Netherlands. E-mail: andre.hidalgo@wur.nl

DOI: 10.1534/g3.115.018119

Table S1 Number of records and individuals used to estimate genetic correlations for purebred and crossbred performance for the four traits under study.

Trait	Population	# records	# individuals
AFI	DL	173,742	173,742
	LW	227,212	227,212
	F1	191,657	191,657
TNB	DL	757,445	176,188
	LW	1,017,925	234,505
	F1	809,746	195,766
LBW	DL	142,510	37,554
	LW	101,634	28,530
	F1	9,293	2,424
LVR	DL	142,510	37,554
	LW	101,634	28,530
	F1	9,293	2,424

DL - Dutch Landrace, LW - Large White, F1 - cross between DL and LW

AFI- age at first insemination, TNB- total number of piglets born, LBW- litter birth weight, LVR- litter variation

Table S2 Estimated pedigree-based heritability (h^2) of the deregressed estimated breeding values across traits and populations under study.

TRAIT	Heritability (S.E.)		
	DL	LW	F1
AFI	0.27 (0.05)	0.22 (0.05)	0.73 (0.16)
TNB	0.03 (0.01)	0.05 (0.01)	0.09 (0.04)
LBW	0.78 (0.09)	0.70 (0.07)	0.43 (0.15)
LVR	0.20 (0.04)	0.12 (0.03)	0.17 (0.08)

DL - Dutch Landrace, LW - Large White, F1 - cross between DL and LW, S.E. – standard error, AFI- age at first insemination, TNB- total number of piglets born, LBW- litter birth weight, LVR- litter variation

Table S3 GEBV accuracies from prediction of crossbred genetic merit from purebred training data using GBLUP (scenarios 12-17) – MOST 50% related animals between training and validation populations

			N training		N prediction	Accuracy	
Trait	Scenario	r^2	DL	LW	F1	GBLUP^{cv}	Bias*
AFI	12	0.45	1067	1389	144	0.05	0.49
TNB	12	0.47	1066	1383	140	0.33	1.93
LBW	12	0.79	1070	1385	143	0.34	0.67
LVR	12	0.52	1069	1385	143	0.24	0.81

DL - Dutch Landrace, LW - Large White, F1 - cross between DL and LW

AFI- age at first insemination, TNB- total number of piglets born, LBW- litter birth weight, LVR- litter variation

^{cv} - Estimate obtained by 20-random training-prediction populations

* - Regression coefficient of the GEBV on the DEBV

r^2 - Mean reliability of deregressed estimated breeding values from the training population

Table S4 GEBV accuracies from prediction of crossbred genetic merit from purebred training data using GBLUP (scenarios 12-17) – LEAST 50% related animals between training and validation populations

Trait	Scenario	r^2	N training		N prediction	Accuracy	Bias*
			DL	LW	F1	GBLUP[cv]	
AFI	12	0.45	1067	1389	144	-0.18	-2.34
TNB	12	0.47	1066	1383	140	0.09	0.80
LBW	12	0.79	1070	1385	143	0.32	0.66
LVR	12	0.52	1069	1385	143	0.35	1.19

DL - Dutch Landrace, LW - Large White, F1 - cross between DL and LW

AFI- age at first insemination, TNB- total number of piglets born, LBW- litter birth weight, LVR- litter variation

[cv] - Estimate obtained by 20-random training-prediction populations

* - Regression coefficient of the GEBV on the DEBV

r^2 - Mean reliability of deregressed estimated breeding values from the training population

Files S1-S3

Available for download as Excel files at www.g3journal.org/lookup/suppl/doi:10.1534/g3.115.018119/-/DC1

File S1 Deregressed EBVs of the Dutch Landrace animals

File S2 Deregressed EBVs of the F1 animals

File S3 Deregressed EBVs of the Large White animals

File S4

G matrix for all individuals across populations.

Available for download at http://figshare.com/articles/File_S4/1425093
