# Supplementary material for: Accuracy of Predicted Genomic Breeding Values in Purebred and Crossbred Pigs
Source: G3 (Bethesda). 2015 May 26;5(8):1575–83. doi: 10.1534/g3.115.018119 (PMC4528314; doi:10.1534/g3.115.018119)
Supplement: Supporting Information [file supp_g3.115.018119_TableS1.pdf]

Table S1 Number of records and individuals used to estimate genetic correlations for purebred and crossbred performance for the four traits under study.

Trait	Population	# records	# individuals
AFI	DL	173,742	173,742
	LW	227,212	227,212
	F1	191,657	191,657
TNB	DL	757,445	176,188
	LW	1,017,925	234,505
	F1	809,746	195,766
LBW	DL	142,510	37,554
	LW	101,634	28,530
	F1	9,293	2,424
LVR	DL	142,510	37,554
	LW	101,634	28,530
	F1	9,293	2,424

DL - Dutch Landrace, LW - Large White, F1 - cross between DL and LW
AFI- age at first insemination, TNB- total number of piglets born, LBW- litter birth weight, LVR- litter variation
