# Supplementary material for: Accuracy of Predicted Genomic Breeding Values in Purebred and Crossbred Pigs
Source: G3 (Bethesda). 2015 May 26;5(8):1575–83. doi: 10.1534/g3.115.018119 (PMC4528314; doi:10.1534/g3.115.018119)
Supplement: Supporting Information [file supp_g3.115.018119_TableS2.pdf]

Table S2 Estimated pedigree-based heritability (h^2) of the deregressed estimated breeding values across traits and populations under study.

TRAIT	Heritability (S.E.)		
	DL	LW	F1
AFI	0.27 (0.05)	0.22 (0.05)	0.73 (0.16)
TNB	0.03 (0.01)	0.05 (0.01)	0.09 (0.04)
LBW	0.78 (0.09)	0.70 (0.07)	0.43 (0.15)
LVR	0.20 (0.04)	0.12 (0.03)	0.17 (0.08)

DL - Dutch Landrace, LW - Large White, F1 - cross between DL and LW, S.E. – standard error, AFI- age at first insemination, TNB- total number of piglets born, LBW- litter birth weight, LVR- litter variation
