# Supplementary material for: Accuracy of Predicted Genomic Breeding Values in Purebred and Crossbred Pigs
Source: G3 (Bethesda). 2015 May 26;5(8):1575–83. doi: 10.1534/g3.115.018119 (PMC4528314; doi:10.1534/g3.115.018119)
Supplement: Supporting Information [file supp_g3.115.018119_TableS3.pdf]

Table S3 GEBV accuracies from prediction of crossbred genetic merit from purebred training data using GBLUP (scenarios 12-17) – MOST 50% related animals between training and validation populations

			N training		N prediction	Accuracy	
Trait	Scenario	r^2	DL	LW	F1	GBLUP^{cv}	Bias*
AFI	12	0.45	1067	1389	144	0.05	0.49
TNB	12	0.47	1066	1383	140	0.33	1.93
LBW	12	0.79	1070	1385	143	0.34	0.67
LVR	12	0.52	1069	1385	143	0.24	0.81

DL - Dutch Landrace, LW - Large White, F1 - cross between DL and LW

AFI- age at first insemination, TNB- total number of piglets born, LBW- litter birth weight, LVR- litter variation

^{cv} - Estimate obtained by 20-random training-prediction populations

* - Regression coefficient of the GEBV on the DEBV

r^2 - Mean reliability of deregressed estimated breeding values from the training population
