# Supplementary material for: Accuracy of Predicted Genomic Breeding Values in Purebred and Crossbred Pigs
Source: G3 (Bethesda). 2015 May 26;5(8):1575–83. doi: 10.1534/g3.115.018119 (PMC4528314; doi:10.1534/g3.115.018119)
Supplement: Supporting Information [file supp_g3.115.018119_TableS4.pdf]

Table S4 GEBV accuracies from prediction of crossbred genetic merit from purebred training data using GBLUP (scenarios 12-17) – LEAST 50% related animals between training and validation populations

			N training		N prediction	Accuracy	
Trait	Scenario	r^2	DL	LW	F1	GBLUP[cv]	Bias[*]
AFI	12	0.45	1067	1389	144	-0.18	-2.34
TNB	12	0.47	1066	1383	140	0.09	0.80
LBW	12	0.79	1070	1385	143	0.32	0.66
LVR	12	0.52	1069	1385	143	0.35	1.19

DL - Dutch Landrace, LW - Large White, F1 - cross between DL and LW

AFI- age at first insemination, TNB- total number of piglets born, LBW- litter birth weight, LVR- litter variation

[cv] - Estimate obtained by 20-random training-prediction populations

[*] - Regression coefficient of the GEBV on the DEBV

r^2 - Mean reliability of deregressed estimated breeding values from the training population
