# Supplementary material for: Accuracy of Predicted Genomic Breeding Values in Purebred and Crossbred Pigs
Source: G3 (Bethesda). 2015 May 26;5(8):1575–83. doi: 10.1534/g3.115.018119 (PMC4528314; doi:10.1534/g3.115.018119)
Supplement: Supporting Information [file supp_g3.115.018119_FileS4.pdf]

File S4

G matrix for all individuals across populations.

Available for download at http://figshare.com/articles/File_S4/1425093
